# Mechanical Response and Functional Performance of Heat-Treated LPBF NiTi Shape Memory Alloys

**DOI:** 10.3390/ma19030627

**Published:** 2026-02-06

**Authors:** Jerzy Ratajski, Błażej Bałasz, Agnieszka Peła, Paweł Krupski, Kamil Bochenk, Michał Tacikowski, Łukasz Major

**Affiliations:** 1 Faculty of Mechanical and Power Engineering, Koszalin University of Technology, 75-453 Koszalin, Poland; blazej.balasz@tu.koszalin.pl (B.B.); agnieszka.pela@tu.koszalin.pl (A.P.); pawel.krupski@tu.koszalin.pl (P.K.); 2 Institute of Fundamental Technological Research, Polish Academy of Sciences, 02-106 Warsaw, Poland; kboch@ippt.pan.pl; 3 Faculty of Materials Science and Engineering, Warsaw University of Technology, 02-507 Warsaw, Poland; michal.tacikowski@pw.edu.pl; 4 Institute of Metallurgy and Materials Engineering, Polish Academy of Sciences, 30-059 Krakow, Poland; l.major@imim.pl

**Keywords:** NiTi shape memory alloy, laser powder bed fusion (LPBF), stress-induced martensite (SIM), pseudoelasticity, aging heat treatment, Ni_4_Ti_3_ precipitation, microstructural homogenization, low-temperature mechanical behavior

## Abstract

**Highlights:**

**What are the main findings?**

- Solution treatment fully restores pseudoelasticity in LPBF NiTi.
- Aging stabilizes martensite via dense Ni_4_Ti_3_ precipitation.
- Deformation modes shift from reversible to irreversible with processing.

**What are the implications of the main findings?**

- Post-processing allows precise tuning of NiTi functional behavior.
- ST enables actuator-type energy-storage applications.
- Aged states enhance energy dissipation and structural strengthening.

**Abstract:**

This study evaluates how solution treatment and aging influence the deformation mechanisms, phase transformations and functional performance of NiTi alloys produced by laser powder bed fusion (LPBF). Tensile tests performed at room temperature (RT) and −20 °C (LT) were combined with Differential Scanning Calorimetry (DSC), X-ray Diffraction (XRD) and Transmission Electron Microscopy (TEM) analyses to correlate mechanical response with transformation thermodynamics and microstructural evolution. In the as-fabricated (AF) condition, deformation is governed by twinning and martensitic plasticity due to suppressed stress-induced martensite (SIM). Solution treatment (ST) restores reversible SIM at RT and preserves partial recoverability at LT as a result of microstructural homogenization and internal stress relief. Aging at 500 °C (A1h, A20h) promotes Ni_4_Ti_3_ precipitation, increasing transformation temperatures and stabilizing martensite, which leads to entirely irreversible deformation at both temperatures. These findings establish a clear functional continuum—ranging from recoverable (ST) to dissipative (AF) and fully irreversible (A20h) behavior—and provide a mechanistic framework for tailoring LPBF NiTi components for actuators, energy-storage and energy-dissipation applications.

## 1. Introduction

NiTi shape memory alloys (SMAs) are widely recognized for their unique ability to exhibit pseudoelasticity, the shape memory effect and temperature-dependent reversible martensitic transformations, primarily between the high-symmetry B2 austenite and the low-symmetry B19′ martensite phases [1,2,3]. Owing to these exceptional functional properties, NiTi has found applications in medical devices, aerospace systems, actuators and mechanical energy-absorption components [4,5,6,7].

The rapid development of additive manufacturing (AM), particularly laser powder bed fusion (LPBF), has opened new opportunities for producing NiTi parts with complex geometries and locally tailored functional behavior. However, LPBF-manufactured NiTi commonly exhibits microstructural inhomogeneities, including chemical segregation, steep thermal gradients, melt-pool-induced textures and significant residual stresses [8]. These features may suppress or distort the stress-induced martensitic (SIM) transformation, leading to reduced strain recoverability and degraded pseudoelastic performance in the as-fabricated (AF) condition [9,10,11,12,13,14,15,16,17,18,19].

Post-processing treatments are therefore essential for restoring or tailoring transformation behavior. Solution treatment (ST) typically relieves internal stresses and homogenizes the microstructure, thereby enhancing the reversibility of martensitic transformations. Aging at 400–550 °C induces the precipitation of coherent Ni_4_Ti_3_ particles, which modify transformation temperatures through elastic interaction with the matrix and Ni depletion effects [20]. While such precipitation strengthening can be beneficial for controlling thermomechanical response, it often reduces pseudoelasticity by stabilizing the martensitic phase.

Although the effects of LPBF processing parameters and post-processing on room-temperature pseudoelasticity have been widely explored [21,22,23,24,25], significantly less attention has been devoted to deformation mechanisms at temperatures below room temperature, where thermodynamic driving forces and microstructural constraints are fundamentally altered. In particular, how ST and aging modify the balance between SIM, twinning and martensitic plasticity under low-temperature (LT) loading remains insufficiently understood. Furthermore, the interaction between Ni_4_Ti_3_ precipitates, local stress fields and strain partitioning during LT deformation has not yet been systematically examined.

Existing studies predominantly address room-temperature behavior and focus on either transformation thermodynamics or mechanical performance, but rarely integrate thermal, structural and mechanical evidence to provide a unified interpretation across the full spectrum of LPBF processing states. The combined effect of ST and aging on phase stability, SIM activation and strain recoverability at both room- and low-temperature conditions remains a clear gap in the literature.

This work provides a comprehensive assessment of how solution treatment and aging (1 h and 20 h at 500 °C) affect microstructure, transformation behavior and mechanical response of LPBF NiTi at 25 °C and −20 °C. By correlating DSC, XRD, TEM and tensile results, we identify the mechanisms governing reversible and irreversible deformation across all processing conditions.

The study offers a unified mechanistic interpretation of LPBF NiTi that spans all major states—AF, ST, A1h and A20h—and two testing temperatures. We demonstrate how microstructural evolution dictates whether deformation is reversible (via SIM), partially recoverable (via mixed SIM and twinning) or fully irreversible (via martensitic plasticity). The work establishes a functional continuum useful for designing LPBF NiTi components for applications requiring mechanical energy storage (actuators) or mechanical energy dissipation (protective structures).

## 2. Materials and Methods

### 2.1. Material and LPBF Processing

**Figure 1:**
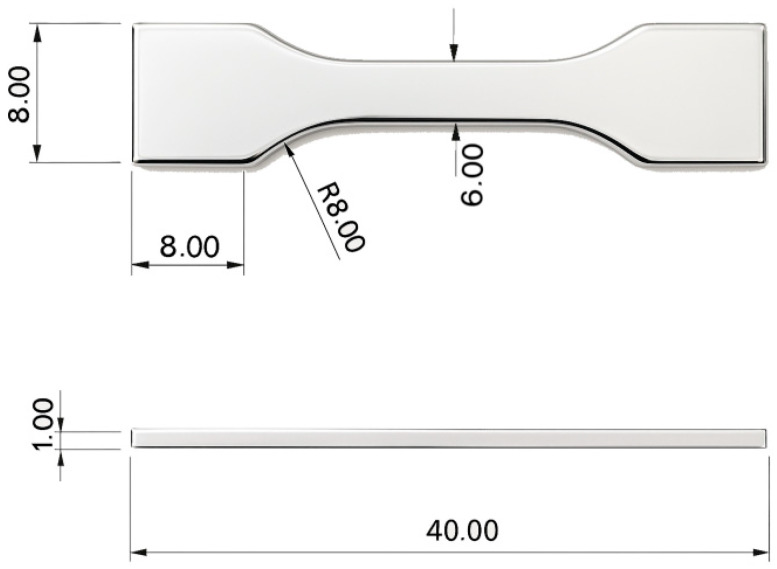
Tensile test specimens with a dog-bone geometry.

Dog-bone tensile specimens (gauge length 40 mm, width 8 mm, thickness 1 mm; Figure 1) were fabricated using laser powder bed fusion (LPBF) from pre-alloyed NiTi powder with a nominal composition of 50.8 at.% Ni and 49.2 at.% Ti.

**Figure 2:**
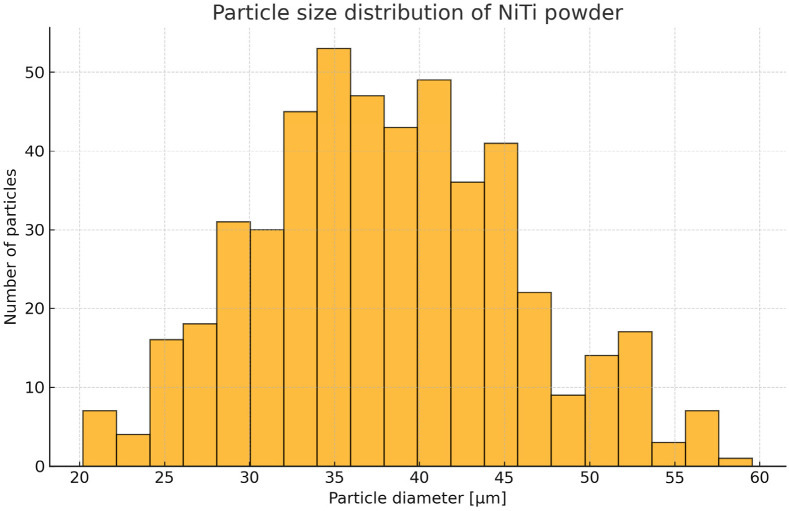
Particle size distribution of NiTi powder presented as a histogram, showing the number of particles as a function of their diameter.

The powder particles exhibited predominantly spherical morphology and a narrow size distribution of 20–60 µm, with the most frequent size between 35 and 40 µm (Figure 2). Approximately 90% of all particles were smaller than 55 µm, confirming good flowability and morphological uniformity of the feedstock.

LPBF processing was carried out using an ORLAS CREATOR (O.R. Lasertechnologie GmbH, Dieburg, Germany) system under a high-purity argon atmosphere with an oxygen content maintained below 0.1%. The low oxygen level minimized oxidation and ensured stable melt-pool conditions. All samples were built in a vertical orientation to reduce thermal distortion and obtain a more uniform microstructure along the build direction. The key processing parameters were as follows (Table 1):

- Laser power: 186 W;
- Scanning speed: 1100 mm/s;
- Hatch spacing: 80 µm;
- Layer thickness: 30 µm;
- Build platform temperature: room temperature;
- Scan strategy: 90° rotation between layers.

These parameters correspond to an energy input commonly used for NiTi and known to produce dense material but with characteristic melt-pool boundaries and residual stresses. The as-built samples are referred to as AF.

The selected LPBF parameters were previously optimized for this specific dog-bone geometry in our earlier study [25], where the combination of 186 W laser power and 1100 mm/s scanning speed yielded fully dense material with stable melt-pool dynamics. This justifies their use in the present work.

### 2.2. Heat Treatments

Two post-processing routes were applied to modify phase stability and microstructural homogeneity.

Solution treatment (ST):

- Heating: 950 °C;
- Holding time: 15 min;
- Quenching: water quench;
- Purpose: removal of residual stresses, dissolution of melt-pool chemical gradients, homogenization of the B2 matrix.

Aging treatments:

Aging was performed to induce Ni_4_Ti_3_ precipitation and tune transformation temperatures.

- Temperature: 500 °C.
- Aging times: 1 h (A1h) and 20 h (A20h).
- Cooling: furnace cooling to room temperature.
- Purpose: precipitation of coherent Ni_4_Ti_3_ particles that modify local internal stresses and increase transformation temperatures.

Longer aging time (20 h) is expected to produce a higher precipitate density and consequently a stronger stabilizing effect on martensite.

### 2.3. DSC Measurement Procedure

Martensitic and austenitic transformation temperatures were determined as follows, using the standard nomenclature commonly adopted in the shape-memory alloy literature:

- Ms—temperature at which the martensitic transformation starts during cooling;
- Mf—temperature at which the martensitic transformation finishes;
- As—temperature at which the austenite begins to form during heating;
- Af—temperature at which the austenite transformation finishes.

Transformation temperatures (Ms, Mf, As, Af) were measured using a NETZSCH DSC 214 calorimeter (NETZSCH-Gerätebau GmbH, Selb, Germany). Approximately 25 mg samples were cycled between −70 °C and 150 °C under a nitrogen atmosphere to prevent oxidation. Heating and cooling rates were maintained at 10 °C/min.

Instrument calibration was performed using the correction file. The equipment sensitivity range was ±5000 µV, with an exothermic signal defined as positive (+1). Measurements were carried out in aluminum Concavus crucibles with perforated lids.

To ensure reproducible measurement conditions, each specimen was subjected to two consecutive DSC cycles. The transformation temperatures reported in this work were extracted from the first cooling and the second heating segments, which is standard practice for NiTi alloys to avoid transient effects from the initial thermal cycle.

DSC data were analyzed to evaluate transformation temperatures, peak sharpness, and transformation pathways (B2 ↔ B19′ ± R-phase) of the sample resulting from LPBF processing and subsequent heat treatments.

### 2.4. XRD Measurem Transmission Electron Microscopy Measurements (TEM) Conditions

Phase identification and qualitative phase-fraction analysis were conducted using an Empyrean Malvern Panalytical diffractometer equipped with Cu Kα radiation (λ = 1.5406 Å). Diffraction patterns were collected in the 2θ range of 20–90° with a step size of 0.02°. All measurements were performed at room temperature under three conditions:

- In the unloaded state;
- Immediately after tensile deformation (within 5 min);
- At room temperature.

The obtained diffraction data enabled evaluation of the amount of retained martensite, the reversibility of the B2 ↔ B19′ transformation, as well as the influence of Ni_4_Ti_3_ precipitates on phase stability.

### 2.5. Transmission Electron Microscopy Measurements (TEM)

Microstructural analysis was performed using a JEOL 2100F TEM (JEOL, Tokyo, Japan) operating at 200 kV. Thin foils were prepared by twin-jet electropolishing in a methanol/nitric acid solution at −20 °C to minimize thermal artifacts.

High-resolution TEM (HRTEM) analysis was used to achieve the following:

- Confirm the crystallographic structure of Ni_4_Ti_3_ precipitates;
- Assess their morphology, size and coherence;
- Examine matrix–precipitate interface features.

Special attention was given to aged samples to evaluate precipitate density and the extent of microstructural evolution.

### 2.6. Tensile Testing

Uniaxial tensile tests were conducted using an Instron 5969 testing machine (Instron, Norwood, MA, USA) at a constant strain rate of 1 × 10^−4^ s^−1^. For each processing condition (AF, ST, A1h, A20h), three specimens (n = 3) were tested to ensure reproducibility.

Two test temperatures were applied:

- Room temperature (RT): 25 °C;
- Low temperature (LT): −20 °C.

LT tests were performed inside a temperature-controlled environmental chamber (stability ± 0.2 °C). The curves shown in Section 3 are representative of three tested specimens for each processing condition.

Stress–strain curves were recorded both during loading and unloading to quantify the following:

- Recoverable strain;
- Irreversible strain;
- Presence or absence of SIM plateaus;
- Differences in hardening behavior between processing conditions.

### 2.7. Sample Labeling

For clarity and consistency, all samples are labeled as follows (Table 2):

## 3. Results

### 3.1. Differential Scanning Calorimetry (DSC)

**Figure 3:**
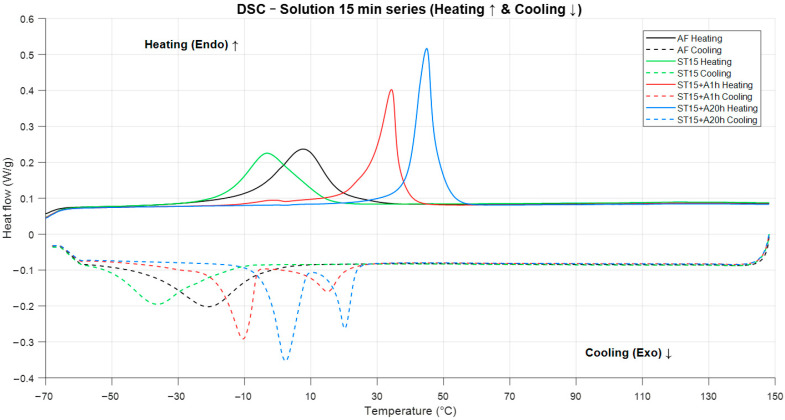
Heating and cooling DSC curves for NiTi samples after 15 min solution treatment (ST) and subsequent aging at various times. The curves show the evolution of transformation peaks corresponding to the reverse (M→A) transformation during heating and the forward (A→M) transformation during cooling. The arrows indicate the direction of heating and cooling.

Figure 3 presents the DSC heating and cooling curves for AF, ST, A1h and A20h samples. A clear and systematic evolution of transformation behavior is observed as a function of post-processing.

The as-fabricated (AF) sample shows the broadest and least defined transformation peaks, indicating a broadened and heterogeneous B2→B19′ transformation response typical of microstructurally heterogeneous LPBF NiTi. The broad peaks and relatively elevated transformation start temperatures are consistent with the presence of significant internal stresses and local chemical/compositional variations, as reported for LPBF NiTi exhibiting heterogeneous microstructure and Ni evaporation–induced composition shifts [26,27].

After solution treatment (ST), the transformation peaks become sharper and show a slight shift toward lower temperatures relative to the AF condition. This sharpening reflects microstructural homogenization and relief of internal stresses. The reduction in peak width indicates the restoration of a more uniform thermodynamic environment for the martensitic transformation.

Aging at 500 °C produces a progressive increase in transformation temperatures. The A1h specimen exhibits narrower and more intense peaks, suggesting increased transformation coherence due to the formation of Ni_4_Ti_3_ precipitates. A weak trace of the R-phase may be present during cooling, although its contribution is minor.

The A20h sample displays the highest transformation temperatures and the most sharply defined peaks. During cooling, a two-step A→R→M transformation is observed, indicating the presence of the R-phase. During heating, the transformation proceeds predominantly through a single B19′→B2 peak. These features are characteristic of strong martensite stabilization caused by a high density of Ni_4_Ti_3_ precipitates.

Overall, the DSC results demonstrate that heat treatment progressively alters transformation pathways by reducing microstructural heterogeneity (ST) or increasing internal stresses through precipitation (A1h, A20h).

### 3.2. X-Ray Diffraction (XRD)

**Figure 4:**
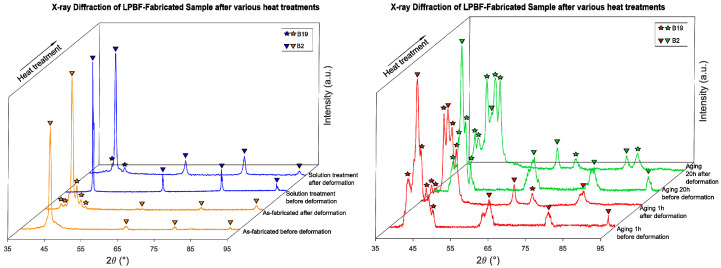
XRD of NiTi samples: in the as-fabricated condition (AF), after solution treatment (ST), after 1 h of aging (A1h) and after 20 h of aging (A20h) before and after deformation.

Figure 4 shows XRD patterns collected before and after tensile deformation for all processing conditions. The results confirm the DSC trends regarding phase stability and transformation reversibility.

In the AF state, the diffraction pattern shows only the B2 austenite phase prior to loading. The peaks are noticeably broader and less well defined than in the heat-treated conditions, reflecting microstructural heterogeneity and residual stresses typical of LPBF NiTi. After solution treatment, the peaks become sharper and better separated, allowing clearer phase identification.

After deformation, AF becomes almost fully martensitic, and the martensite does not revert back to B2 upon unloading, indicating irreversible deformation dominated by martensitic plasticity.

The ST sample exhibits a predominantly B2 structure before loading, demonstrating successful stress relief and structural homogenization. After deformation at RT, B19′ peaks appear, confirming the activation of stress-induced martensite (SIM). After unloading, the XRD patterns show a partial recovery of the B2 phase, indicating that a portion of the stress-induced martensite has reverted to austenite. This observation reflects the post-unloading retained phase state and correlates with the pseudoelastic recovery observed in tensile tests. This result correlates well with the pseudoelastic behavior observed in tensile tests.

The A1h and A20h samples show increasing fractions of B19′ prior to loading, reflecting elevated transformation temperatures and partial martensite stabilization resulting from Ni_4_Ti_3_ precipitation. After deformation, both aged conditions retain significant amounts of martensite. The retained B19′ fraction increases with aging time, with A20h showing the strongest stabilization and minimal reversibility.

These results confirm that the presence, density and distribution of Ni_4_Ti_3_ precipitates dictate the extent of martensite stabilization and the reversibility of deformation. These effects are consistent with the expected formation of Ni_4_Ti_3_ precipitates during aging, which is confirmed by TEM observations presented in Section 3.3.

### 3.3. Transmission Electron Microscopy (TEM)

**Figure 5:**
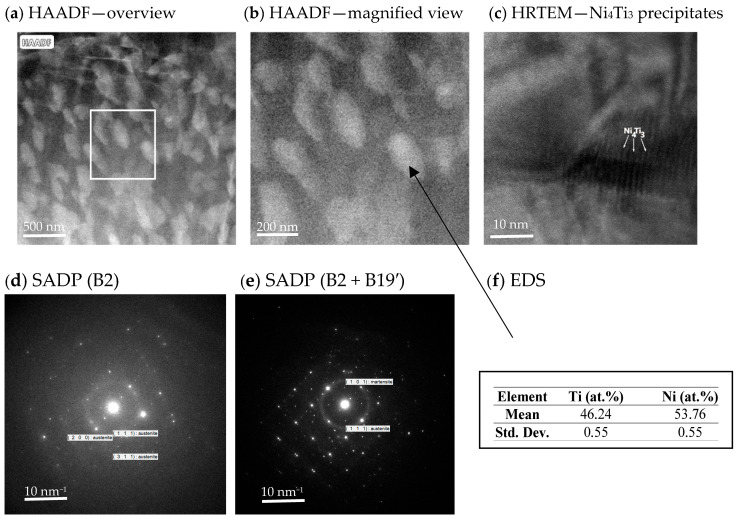
Microstructure of the NiTi alloy after Laser Powder Bed Fusion (LPBF) and aging at 500 °C for 20 h: (**a**) HAADF overview image showing a uniform distribution of bright Ni_4_Ti_3_ precipitates within the B2 matrix; (**b**) HAADF magnified view of the selected region in (**a**), highlighting the plate-like morphology and density of the precipitates; (**c**) HRTEM image with marked Ni_4_Ti_3_ precipitates; (**d**) SAED pattern acquired from the matrix region, indexed with characteristic B2 reflections ({111}, {200}, {220}); (**e**) SAED pattern acquired from a region containing precipitates and local martensite variants, showing additional B19′ reflections ({101}) together with B2 reflections; (**f**) EDS results measured from the bright plate-like precipitates, confirming their Ni-rich composition consistent with Ni_4_Ti_3_.

Figure 5 presents the High-Angle Annular Dark-Field Scanning Transmission Electron Microscopy (HAADF–STEM), High-resolution transmission electron microscopy (HRTEM) and Selected area electron diffraction (SAED) analysis of the NiTi alloy aged at 500 °C for 20 h (A20h). The microstructure is dominated by a dense population of Ni_4_Ti_3_ precipitates coherently embedded within the B2 matrix.

At low magnification (Figure 5a), the precipitates appear uniformly distributed throughout the microstructure. Their bright contrast is characteristic of Ni-rich phases, confirming compositional partitioning associated with long-term aging [28]. The homogeneity of the precipitate distribution indicates diffusion-controlled precipitation without localized clustering.

Higher magnification (Figure 5b) reveals the typical plate-like morphology of Ni_4_Ti_3_ precipitates and clearly defined precipitate–matrix interfaces. The spatial arrangement and size of these precipitates are consistent with advanced stage aging and correlate directly with the significant increase in transformation temperatures observed in DSC [29,30].

The HRTEM image (Figure 5c) identifies the Ni_4_Ti_3_ precipitates, while the corresponding SAED patterns (Figure 5d,e) provide crystallographic confirmation of the matrix and secondary phases. The SAED pattern from the matrix region shows reflections indexed as B2 ({111}, {200}, {220}), whereas the pattern obtained from regions containing precipitates exhibits additional reflections corresponding to B19′ martensite ({101}). These diffraction results confirm the presence of both B2 and B19′ phases in the aged microstructure.

Although the HRTEM contrast suggests lattice alignment consistent with a semi-coherent interface, the available resolution does not allow definitive confirmation of interface coherency. The EDS analysis (Figure 5f) demonstrates the Ni-rich composition of the plate-like precipitates, fully consistent with Ni_4_Ti_3_.

The aged A20h sample exhibits a very high density of plate-like Ni_4_Ti_3_ precipitates, which is known to induce significant lattice distortions and promote the stabilization of martensite, as widely reported in the literature [31,32]. Although the present TEM analysis does not allow direct measurement of internal stress fields (e.g., via GPA), the observed precipitate morphology and density are consistent with the strong martensite retention detected in XRD and with the irreversible deformation behavior recorded during mechanical testing. The large population of Ni_4_Ti_3_ precipitates therefore suppresses SIM and favors deformation dominated by martensitic plasticity rather than reversible transformation.

### 3.4. Tensile Behavior at Room Temperature (RT)

**Figure 6:**
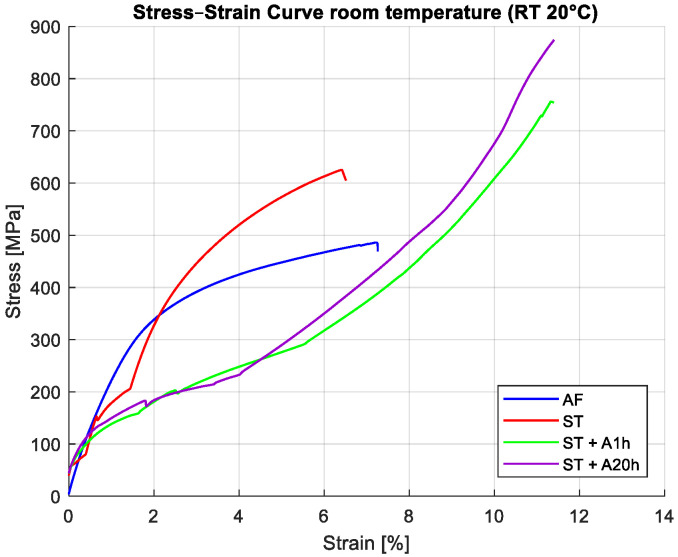
Room-temperature (RT) stress–strain curves for samples subjected to different heat treatments, illustrating distinct pseudoelastic behavior and strength levels.

Representative stress–strain curves at RT are shown in Figure 6. Distinct deformation behavior is observed for each processing condition.

The AF sample exhibits a nearly linear elastic response followed by gradual strain hardening without a clear SIM plateau. This behavior indicates suppression of SIM and dominance of twinning and martensitic plasticity [33].

The ST sample shows a well-defined stress-induced martensite plateau, followed by a stable pseudoelastic response and nearly complete strain recovery upon unloading. This confirms that solution treatment restores reversible SIM and pseudoelasticity.

The A1h and A20h samples show continuous hardening throughout deformation, with no evidence of a pseudoelastic plateau. Strength increases with aging time, and recoverable strain decreases. The A20h condition exhibits the highest stiffness and most pronounced irreversibility due to strong martensite stabilization.

### 3.5. Tensile Behavior at Low Temperature (LT)

**Figure 7:**
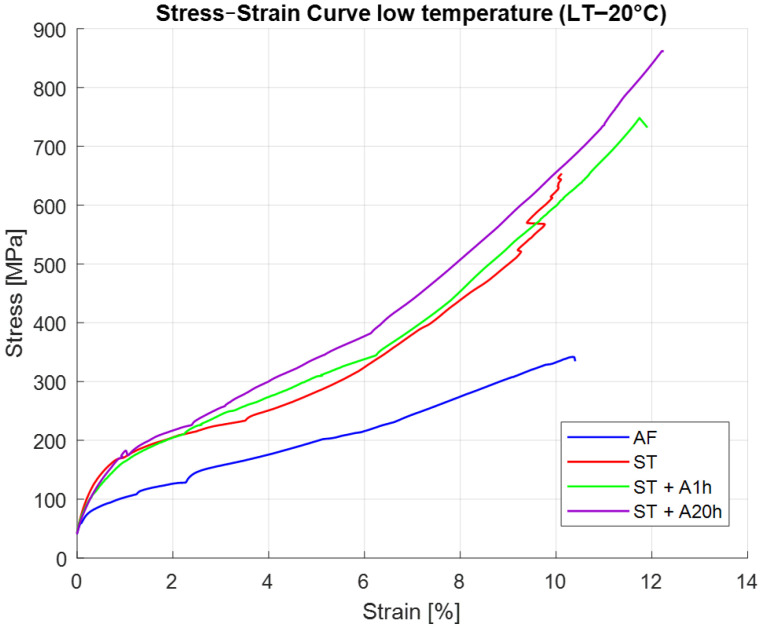
Tensile curves at −20 °C (LT) for samples subjected to different heat treatments, showing fully martensitic behavior in AF, limited recoverability in ST, and increasing strength and hardening in aged conditions.

Figure 7 compares tensile responses at −20 °C. The AF specimen is fully martensitic at LT and deforms by monotonic hardening without any pseudoelastic features. The absence of a plateau indicates purely martensitic plasticity.

The ST sample shows an initial nonlinear response, followed by limited strain recovery upon unloading. This is consistent with partial SIM activity, because a fraction of the material remains in the B2 phase near −20 °C, allowing some reversible transformation.

Both A1h and A20h samples deform by purely martensitic mechanisms at LT. The hardening rate and strength increase with precipitation density, and no recoverable strain is observed. This confirms that precipitation-induced martensite stabilization suppresses SIM at sub-zero temperatures [9,34]. The microstructural evolution during unloading was assessed not from the tensile curves alone, but from the post-unloading XRD measurements, which directly quantify the extent of B19′→B2 reversion.

## 4. Discussion

The DSC results (Figure 3) clearly demonstrate that post-processing significantly affects the thermodynamics of the B2↔B19′ transformation. The AF condition shows broad, weakly defined peaks, which are typical of LPBF NiTi containing strong residual stresses, chemical gradients and melt-pool heterogeneity. These factors broaden the thermodynamic distribution of transformation temperatures [15,33].

Solution treatment homogenizes the chemical composition and relieves internal stresses, producing sharper and more coherent transformation peaks. The reduction in peak breadth indicates a more uniform driving force for martensitic transformation.

Aging at 500 °C produces a progressive increase in transformation temperatures due to the formation of Ni_4_Ti_3_ precipitates. These precipitates locally deplete Ni in the matrix, increasing its effective Ti content and thereby raising Ms and Af [29]. Additionally, precipitate-induced internal stress fields stabilize the martensite phase [35], which is particularly evident in the A20h condition [30].

Overall, post-processing introduces a clear shift from a stress-dominated (AF), through a homogenized (ST), to a precipitate-stabilized state (A1h, A20h), each with distinct thermodynamic signatures.

The mechanical response of the AF samples at both RT and LT is dominated by irreversible mechanisms. The absence of a SIM plateau, combined with monotonic hardening, indicates that twinning and martensitic plasticity—rather than reversible SIM—govern deformation [19,36,37].

The XRD patterns support this interpretation: although only the B2 austenite phase is detectable in the AF samples before loading, the strong peak broadening and overlap characteristic of LPBF-produced microstructures limit phase resolution and may obscure weak B19′ contributions. After deformation, they retain high fractions of B19′, with little or no reversion to B2 upon unloading. This suggests insufficient mobility of transformation fronts, which is consistent with the presence of melt-pool boundaries, compositional gradients and nanoscale heterogeneity documented in prior LPBF NiTi studies [10,11,12,13,14,15,16,17,18,19].

Thus, the AF condition exhibits limited functional behavior and acts primarily as a dissipative material, unsuitable for pseudoelastic applications but potentially relevant for energy absorption.

Solution treatment dramatically improves functional performance, especially at RT. The ST sample shows a well-defined SIM plateau and nearly complete strain recovery upon unloading, confirming that SIM propagation becomes reversible once internal stress fields and microchemical gradients are removed.

The XRD patterns after deformation show partial restoration of the B2 phase, and DSC reveals sharper peaks compared to AF, confirming improved phase reversibility. These observations are consistent with prior findings showing that ST is a critical step for restoring pseudoelasticity in AM NiTi [17,23].

At LT, ST retains partial pseudoelasticity. Although the alloy is closer to Ms at −20 °C and a portion of the microstructure is martensitic before loading, the remaining B2 fraction is sufficient for limited SIM. This partially recoverable behavior aligns well with the XRD observations, which show mixed B2/B19′ after deformation and partial reversion on unloading.

Thus, ST represents the most functional condition, capable of energy storage via reversible SIM.

Aging produces Ni_4_Ti_3_ precipitates that modify transformation temperatures and deformation mechanisms. The TEM results (Figure 5) show a dense population of coherent plate-like precipitates in A20h, providing direct microstructural evidence of advanced precipitation.

The mechanical response of the aged NiTi samples is strongly governed by the presence of Ni_4_Ti_3_ precipitates. These particles generate coherency strains that modify the local lattice environment, promoting martensite stabilization and reducing the propensity for stress-induced martensite formation. Simultaneously, Ni depletion in the surrounding matrix increases the transformation temperatures (Ms and Af), altering the functional response of the alloy. The precipitates also act as effective obstacles to dislocation motion, resulting in increased yield strength [32,33,38]. As a consequence, the aged samples exhibit no SIM plateau and deform through monotonic hardening, displaying elevated strength but minimal or fully absent strain recovery.

The A20h sample exhibits the strongest stabilization due to its high precipitate density, consistent with the sharpest DSC peaks and highest transformation temperatures.

Thus, aging transforms LPBF NiTi into a structurally strengthened but non-functional state, where martensitic plasticity dominates.

Temperature significantly alters the balance between SIM, twinning and plasticity across all processing conditions [9,34,37].

At room temperature (25 °C), the ST condition demonstrates full pseudoelastic behavior, whereas the AF material exhibits only limited SIM before transitioning into plastic deformation. The aged states (A1h and A20h), stabilized by dense populations of Ni_4_Ti_3_ precipitates, deform predominantly through martensitic plasticity without reversible transformation. At low temperature (−20 °C), the AF sample becomes fully martensitic and deforms plastically, while the ST material retains a small fraction of B2 austenite, enabling limited SIM activity. The aged samples remain martensitic at −20 °C and therefore display fully irreversible deformation.

Lower temperature amplifies differences between conditions, highlighting how processing history governs phase stability and available deformation pathways.

Integrating the mechanical, thermal and structural results reveals a clear functional continuum for LPBF NiTi, summarized in Table 3.

This continuum of deformation mechanisms provides a practical framework for selecting LPBF NiTi conditions for targeted applications. Solution-treated material (ST) is suitable for actuators or energy storage elements where reversible SIM is required. The as-fabricated condition (AF) is advantageous for damping or energy-dissipation applications due to its combined twinning and plasticity response. The extensively aged condition (A20h), characterized by high strength and irreversible deformation, is most appropriate for structural reinforcement or load-bearing components.

Such a unified interpretation, covering all relevant processing states and temperatures, is not present in prior literature and constitutes a key contribution of this work.

Several limitations should be considered when interpreting the present results. The study investigates only one LPBF parameter set, and alternative laser strategies could modify melt-pool morphology and microstructural heterogeneity. Cyclic loading tests were not performed, leaving functional fatigue behavior unexamined. Furthermore, only two aging durations were evaluated; intermediate times may provide additional insight into the relationship between precipitate evolution and mechanical response. The XRD analysis presented here is qualitative, and quantitative determination of phase fractions would enhance the conclusions. Finally, in situ characterization techniques such as synchrotron XRD or digital image correlation could offer direct insight into the kinetics of stress-induced martensite formation and variant evolution.

Despite these constraints, the correlations between DSC, XRD, TEM and mechanical data provide a robust and coherent picture of how post-processing controls the functional response of LPBF NiTi.

## 5. Conclusions

This study evaluates how solution treatment and aging at 500 °C influence the thermomechanical and functional behavior of LPBF-produced Ni-rich NiTi by combining DSC, XRD, TEM and tensile tests at 25 °C and −20 °C.

1. Solution treatment (ST) restores pseudoelasticity by relieving LPBF-induced residual stresses and chemical gradients, producing sharper transformation peaks and a predominantly B2 structure. ST specimens show a clear SIM plateau and near-full strain recovery at room temperature, while still exhibiting partial pseudoelasticity at −20 °C.
2. Aging suppresses SIM and stabilizes martensite through Ni_4_Ti_3_ precipitation. Aging at 500 °C generates Ni_4_Ti_3_ precipitates that raise transformation temperatures and stabilize martensite. Both aged states harden continuously, lack a pseudoelastic plateau, and deform irreversibly.
3. Mechanical behavior evolves with processing state: AF material is governed by twinning and martensitic plasticity, ST enables reversible SIM, while aging (especially A20h) eliminates recoverable strain and results in fully martensitic plasticity across temperatures.
4. Low-temperature loading amplifies differences between states. At −20 °C, AF and aged samples deform entirely through martensitic mechanisms, whereas ST retains limited SIM due to the presence of residual austenite.
5. Microstructural evolution controls functional response: homogenization promotes reversible transformation, whereas precipitation stabilizes martensite and shifts behavior toward irreversibility, consistent with DSC, XRD and TEM findings.

A processing-dependent functional continuum is established:

- ST→pseudoelastic, actuator-capable;
- AF→partially recoverable, energy-dissipative;
- A20h→irreversible, strength-oriented.

This continuum guides the tailoring of LPBF NiTi for applications requiring actuation, energy storage or dissipation, and provides a unified framework linking microstructure, transformation thermodynamics and deformation mechanisms.

## Figures and Tables

**Table 1:** Summary of LPBF processing parameters.

Variable	Value
Laser power	186 W
Scanning speed	1100 mm/s
Hatch spacing	80 µm
Layer thickness	30 µm
Platform temperature	RT
Scan strategy	90° rotation

**Table 2:** Summary of LPBF NiTi sample labeling and corresponding post-processing conditions.

Label	Condition
AF	As-fabricated (LPBF)
ST	Solution-treated (950 °C/15 min/water quench)
A1h	Aged 1 h at 500 °C
A20h	Aged 20 h at 500 °C

**Table 3:** Functional classification of LPBF NiTi samples based on processing condition, recoverability, and dominant deformation mechanisms.

Condition	Functional Behavior	Dominant Mechanisms
ST	recoverable	reversible SIM
AF	partially recoverable/dissipative	twinning + martensitic plasticity
A1h/A20h	fully irreversible	martensitic plasticity + precipitate stabilization

## Data Availability

The data presented in this study are available on request from the corresponding author due to technical and organizational reasons.

## Abbreviations

The following abbreviations are used in this manuscript:

AF	As-fabricated (LPBF) condition
ST	Solution-treated condition
A1h	Aged 1 h at 500 °C
A20h	Aged 20 h at 500 °C
LPBF	Laser Powder Bed Fusion
SMA	Shape Memory Alloy
SIM	Stress-Induced Martensite
DSC	Differential Scanning Calorimetry
XRD	X-ray Diffraction
TEM	Transmission Electron Microscopy
HRTEM	High-Resolution Transmission Electron Microscopy
RT	Room Temperature (25 °C)
LT	Low Temperature (−20 °C)
B2	Austenite phase of NiTi
B19′	Martensite phase of NiTi
R-phase	Intermediate rhombohedral phase
Ni_4_Ti_3_	Precipitate phase formed during aging
HAADF	High-Angle Annular Dark Field
STEM	Scanning Transmission Electron Microscopy
